# Rapid micropropagation and chemical profiling of *in vitro* plantlets and agarwood of *Gyrinops walla* Gaertn. by gas-chromatography and mass-spectrometry

**DOI:** 10.1371/journal.pone.0321049

**Published:** 2025-04-08

**Authors:** S. Selvaskanthan, Lalith Jayasinghe, J. P. Eeswara

**Affiliations:** 1 Department of Agronomy, Faculty of Agriculture, University of Jaffna, Jaffna, Sri Lanka; 2 Postgraduate Institute of Agriculture, University of Peradeniya, Kandy, Sri Lanka; 3 National Institute of Fundamental Studies, Kandy, Sri Lanka; 4 Department of Crop Science, Faculty of Agriculture, University of Peradeniya, Kandy, Sri Lanka; Central University of Punjab, INDIA

## Abstract

*Gyrinops walla* Garten., which is an endemic and endangered species of Sri Lanka, produces the world’s most expensive agarwood used in perfume industry. The high demand for agarwood has resulted in indiscriminate felling of trees, thus threatening the survival of the species. The present study aimed to develop an efficient *in vitro* rapid multiplication technique to conserve the existing trees from extinction, by ensuring the sustainable supply of planting materials for commercial cultivations and to investigate the possibility of producing fragrance compounds by *in vitro* plantlets without felling trees. Efficient micropropagation protocol was developed from axillary buds and shoot tip explants. Murashige and Skoog (MS) medium supplemented with 1.0 mg/L BAP was the best for the establishment of both shoot tips (80.0%) and axillary buds (86.0%). Regenerated buds were further multiplied (10.6 ± 0.93 shoot buds/regenerated shoot) and elongated (4.0 ± 0.26 cm) by transferring to MS medium supplemented with 1.0 mg/L BAP, 0.1 mg/L IBA and 40 g/L sucrose. Highest *in vitro* rooting percentage (66.7%) was recorded in ½  MS medium supplemented with 1.0 mg/L IAA and 40 g/L sucrose. However, none of the shoots rooted on MS media could be acclimatized. Significantly higher percentage of rooted shoots (93.3%) were produced on sand medium without auxin treatment compared to shoots cultured on MS medium supplemented with 1.0 mg/L IAA (66%) and successfully acclimatized with 83.6% survival rate in a medium consisted of sand, topsoil, and compost (1:1:1 ratio). TLC fingerprints of ethyl acetate extracts of *in vitro* grown plantlets and agarwood produced similar spots at the retention factors (R_*f*_) of 0.60, 0.66, and 0.87 under 15% methanol: 85% chloroform solvent system. Chemicals present in *in vitro* plantlets were identified and compared with the agarwood of naturally grown *G. walla* by GC-MS. Both natural agarwood and *in vitro* grown shoot extracts contained 4-Hydroxypyridine 1-oxide (23.2%), 2-tetradecene (16.3%), 1-hexadecene (0.3%), E-15-heptadecenal (19.8%), 18-norabietane (0.6%) and eicosane (0.4%). Present study successfully developed a protocol for rapid multiplication of *G. walla* and indicates the possibility of using of *in vitro* plantlets to produce agarwood resinous compounds.

## Introduction

*Gyrinops walla* Gaertn., belongs to the family Thymelaeaceae, produces fragrant resinous compounds widely used in the perfumery and cosmetic industry [1,2]. It has been protected under the Flora and Fauna Protection Ordinance (Amendment) No 49 of 1993 in Sri Lanka since 2004 and listed in Appendix II of the Convention on International Trade in Endangered Species of Wild Fauna and Flora (CITES), since 2005. The higher international demand for the perfume industry in the Middle East countries has driven large-scale illegal harvesting of the trees from tropical rainforests in Sri Lanka. Extensive exploitation of this species has resulted in enhanced tree mortality, reduced growth rate and the percentage of adult trees that produce seeds [3].

Even though, sexual propagation is a reliable method for *G. walla*, rare seed production, low seed viability, and low germination rate makes it inadequate to meet the current demand of seedling supplies. Furthermore, delayed rooting of seedlings and long life cycle of this plant species are the barriers for conventional propagation under natural conditions [4]. Thus, *in vitro* propagation of *G. walla* would be a better and viable alternative for production of plantlets to fulfil the existing demand.

The use of shoot cultures is another alternative to produce resinous fragrance compounds than the felling of mature trees. Thus, identification of chemicals composition of *in vitro* plantlets of *G. walla* and comparison with the chemicals present in agarwood is necessary before employing tissue culture techniques for product synthesis. Thin Layer Chromatography (TLC) is a versatile method for primary screening of phytochemicals present in any sample. Gas chromatography- Mass spectroscopy (GC-MS) is another useful technique for screening, identification, and quantification of many non-polar and semi-polar volatile compounds. Its high separation power accompanied with a mass spectroscopy detector makes this technique an important tool in the analysis of ultra-trace levels of compounds present in a sample.

Therefore, the present study aimed at rapid multiplication of *G. walla* that would reduce the gap created due to over-exploitation of this species while giving an opportunity to conserve the natural population in the wild habitats. Furthermore, identification and comparison of chemical constituents of *in vitro* plantlets and agarwood provides an opportunity to produce fragrance compounds through shoot cultures of *G. walla* protecting the trees growing in natural ecosystems.

## Materials and methods

### Experimental locations

All the micropropagation studies were conducted at the Department of Crop Science, Faculty of Agriculture, University of Peradeniya while chemical analysis was done at the Natural Product Laboratory, National Institute of Fundamental studies.

### Plant materials

One year old *G. walla* seedlings obtained from the Divisional Forest Office, Ratnapura, Sri Lanka were grown in pots containing the mixture of compost, soil, and sand at a 1:1:1 ratio. Seedlings were kept inside the glasshouse at 27 ± 2 °C and the photoperiod of 12 hours light/12 hours dark at the Agricultural Biotechnology Centre, Faculty of Agriculture, University of Peradeniya, Sri Lanka.

Mother plants were pre-treated with 0.1% w/v of redoxyl metalaxyl (CIC, Sri Lanka) once a week continuously for 4 weeks before collecting the explants to reduce the contamination during the establishment of *in vitro* cultures.

### Establishment of axillary buds and shoot tip cultures

Freshly excised nodal and apical segments of *G. walla* were washed thoroughly under slow running tap water for 10 minutes. The explants were then washed in liquid soap (teepol Lankem, Sri Lanka) for 1 minute and rinsed thoroughly with H_2_O for another 10 minutes.

Then shoot tips and axillary buds were excised and sterilized separately using the procedure reported earlier under a laminar flow cabinet (Labgard Class II, Type A/B3) [5]. The surface sterilized shoot tips and axillary bud explants were trimmed to 1.2–1.5 cm in length and single explant was placed on culture tube containing sterilized (121 °C for 20 minutes at 15 psi) MS [6] medium supplemented with 30 g/L sucrose with four different concentrations (0, 1.0, 2.0 or 5.0 mg/L) of Benzyl Amino Purine. Media pH was adjusted to 5.8 before autoclaving. Then 5–8 growing buds were transferred to the culture jars containing the same media (establishment media) at four week interval regularly until 4^th^ sub-culture. Based on the results of this experiment, MS medium supplemented with 1.0 or 2.0 mg/L BAP was selected for further studies on elongation and multiplication of proliferated shoots.

### *In vitro* multiplication and elongation of established shoots

After 4^th^ subculture (16^th^ weeks after establishment), the multiple shoots were separated from the clusters into single shoots and all the dead tissues were removed. Every single shoot was cut at the basal end and growing shoots were transferred to fresh MS media containing either 30 or 40 g/L of sucrose supplemented with BAP (1.0 or 2.0 mg/L), 0.1 mg/L filter sterilized gibberellic acid (GA_3_) or 1.0 mg/L BAP in combination with 0.1 mg/L indole-3-buteric acid (IBA) (Table 1). Sub-culturing was done regularly every four weeks intervals.

**Table 1:** Influence of different sucrose and PGRs level on growth parameters at multiplication and elongation stage.

MS media containing different PGRs combinations and sucrose				No. of leaves/ shoot	No. of shoots/ cluster	Shoot length (cm)	No. of leaves/ shoot	No. of shoots/ cluster	Shoot length (cm)
Sucrose (g/L)	BAP (mg/L)	IBA (mg/L)	GA_3_ (mg/L)	At first subculture after transferring (at the end of 20^th^ week after establishment)			At second subculture after transferring (at the end of 24^th^ week after establishment)		
30	0.0	0.0	0.0	1.8 ± 0.37c	1.2 ± 0.20c	0.7 ± 0.07c	2.4 ± 0.24d	2.0 ± 0.32c	1.2 ± 0.14f
40	0.0	0.0	0.0	2.2 ± 0.37c	1.4 ± 0.24c	0.8 ± 0.04c	2.8 ± 0.20d	2.4 ± 0.24c	1.6 ± 0.14ed
30	0.0	0.0	0.1	2.8 ± 0.37c	1.4 ± 0.24c	1.0 ± 0.07c	3.2 ± 0.37c	1.4 ± 0.24d	1.7 ± 0.06d
40	0.0	0.0	0.1	4.0 ± 0.45b	2.0 ± 0.32c	1.3 ± 0.22b	3.8 ± 0.58c	2.0 ± 0.32c	2.2 ± 0.11c
30	1.0	0.0	0.0	2.6 ± 0.24c	2.0 ± 0.32c	0.9 ± 0.08c	3.4 ± 0.24c	1.4 ± 0.24d	1.5 ± 0.07e
40	1.0	0.0	0.0	3.8 ± 0.37b	1.8 ± 0.37c	1.3 ± 0.11b	4.0 ± 0.55c	1.4 ± 0.24d	1.9 ± 0.16d
30	2.0	0.0	0.0	1.8 ± 0.37c	1.6 ± 0.24c	0.7 ± 0.07c	1.0 ± 0.45e	0.6 ± 0.2e	0.5 ± 0.22g
40	2.0	0.0	0.0	1.4 ± 0.24c	1.6 ± 0.24c	1.0 ± 0.06c	1.2 ± 0.37e	1.0 ± 0.32b	1.0 ± 0.27f
30	1.0	0.1	0.0	4.2 ± 0.37b	6.4 ± 0.40b	2.0 ± 0.11a	5.4 ± 0.24b	7.2 ± 0.49b	3.1 ± 0.11b
**40**	**1.0**	**0.1**	**0.0**	**7.0 ± 0.71a**	**9.0 ± 0.71a**	**2.1 ± 0.14a**	**8.0f ± 0.55a**	**10.6 ± 0.93a**	**4.0 ± 0.26a**

* Data represented shows mean ±  SE. Data followed by the different letters in a column are significant at *P* ≤ 0.05.

### *In vitro* rooting on MS medium

The effect of different concentrations of IAA, IBA, NAA (0, 0.5, 1.0 and 2.0 mg/L) at 30 or 40 g/L sucrose concentration on rooting was investigated in a preliminary study [7]. The results showed (not included in the present paper) very low success for rooting at all IBA and NAA concentrations [7]. Majority of the shoots grown on IBA and NAA media showed senescence and necrosis. Therefore, the experiment was repeated only with IAA. The shoots elongated and multiplied on MS medium supplemented with 40 g/L of sucrose, 1.0 mg/L BAP and 0.1 mg/L IBA were transferred into ½  MS media containing two sucrose levels (30 or 40 g/L), with two different concentrations (0.5 and 1.0 mg/L) of IAA.

### *In vitro* rooting on sterilized sand medium

The basal ends of the healthy, vigorous shoots originated from the MS medium supplemented with 40 g/L of sucrose, 1.0 mg/L BAP and 0.1 mg/L IBA were dipped in two different concentrations (0 or 1000 mg/L) of IAA or IBA solutions either for 15 or 30 minutes. The treated shoots were established in glass jars (350 mL) containing sterilized sand (nearly 167.5 g) wetted with sterilized distilled water (35 mL). All these procedures were conducted under sterile conditions inside a laminar flow cabinet. Then the bottles were covered with polypropylene sheets and transferred to the culture room. Based on the results obtained for *in vitro* rooting on MS medium, few samples were removed at weekly intervals to observe the rooting process and data obtained at the 8^th^ week after planting was used for analysis.

### Culture conditions

All the cultures were maintained inside glass containers covered with polypropylene sheets maintaining 100% RH inside the culture bottles. Cultures were incubated under cool white fluorescent light (1000 lux), with the photoperiod of 16/8 hours light/dark at 25 ± 2 °C which has shown highly successful in previous studies [8,9]. Temperature of the culture room was maintained using an air conditioner since the laboratory is situated in mid country wet zone of Sri Lanka where average temperature was about 23–28 °C and RH is 75–80%.

### Acclimatization

All the *in vitro* rooted shoots on ½  MS media supplemented with 30 or 40 g/L sucrose and 0.5 or 1.0 mg/L of IAA were transferred to potting mixture consisting of soil, sand and compost in 1:1:1 ratio for acclimatization inside the single propagators prepared by using polypropylene bags. Then the plants were gradually exposed to the outside environment by making holes starting at two weeks after transferring into single propagators.

The sand bottles containing rooted shoots were directly transferred to the plant house and allowed to adapt into outside environment by gradually making small holes on polypropylene cover starting two weeks after transferring to the plant house. Four weeks after transferring to the plant house, the polypropylene cover was removed completely.

The plantlets were allowed to remain in the opened sand bottles or single propagators for another two weeks before transferring into pots consisting of soil, sand and compost in 1:1:1 ratio.

### Data collection

Percentage of explants induced shoot buds, mean length of shoot bud and mean number of shoot buds from shoot tip and axillary budswere recorded at the establishment stage. During elongation and multiplication stage, the number of shoots multiplied per cluster, number of leaves per shoot and shoot length were recorded. Percentage of rooted shoots, number of roots per shoot and mean root length were recorded weekly after eight weeks of transferring shoots into the rooting media.

### Data analysis

Experiments were laid in complete randomized design (CRD). In all the experiments each treatment was replicated five times and each replicate contained five to eight shoots. Parametric data were subjected to analysis of variance (ANOVA). Mean separation was done by Tukey’s test at the 5% level using Minitab 17 statistical software package (minitab.com).

### Phytochemical analysis of *in vitro* plantlets and agarwood of *G. walla* using chromatographic techniques

#### Preparation of extracts for chromatographic studies.

Freeze-dried *in vitro* plantlets and agarwood samples were crushed separately to a fine powder by using a mortar and pestle. The crushed samples were subjected to ultrasound-assisted solvent extraction (ROCKER ultrasonic cleaner, model- soner 206H) for 30 minutes with 100 mL of HPLC grade hexane (Sigma, UK). The extracts were filtered using a Whatman number 1 filter paper and residue was further extracted twice with hexane. Then the solvents collected from three extractions were combined and evaporated to dryness using a rotary evaporator (Heidolph, Laborota 4000) at 90 rpm and temperature below 40 °C, until viscous semi-dried crude extract was obtained. The residual materials were again extracted thrice with HPLC grade dichloromethane (Sigma, UK) and ethyl acetate (Sigma, UK) sequentially and the extracts were evaporated to dryness using a rotary evaporator as described for hexane extracts.

The crude extract (5 mg) obtained after drying was dissolved in l mL of HPLC grade hexane, dichloromethane or ethyl acetate based on the original extracting solution. Then the extracts were filtered using a glass syringe attached with 0.45-micron disposable membrane filters before being injected to GC-MS.

#### Development of TLC fingerprints.

Ethyl acetate has been reported to be the best solvent for extraction for Thin Layer Chromatography of *G. walla* samples based on numbers and intensity of spots [10]. Both hexane and dichloromethane extracts did not produce clear bands on TLC plates [10]. Thus, 5.0 mg/mL of ethyl acetate extracts of *in vitro* plantlets and agarwood were spotted using capillary tubes on TLC silica plates pre-coated on aluminium foil (Merck, 105554, TLC Silica gel 60 F). Then the solvents were allowed to evaporate completely and the spotted plates were placed in a chromatographic solvent chamber containing 15% methanol (Sigma, UK): 85% chloroform (Sigma, UK), which was identified as the best mobile phase in an earlier study [10]. The TLC plates were propped vertically in the solvent chamber and allowed to stand for sufficient time for elution. When the solvent front had nearly reached the top of the stationary phase, the plate was removed from the chamber and the developed TLC plates were air dried. UV monitoring at 254 nm was performed for the detection of the compounds present in the crude extract using UV lamp (VILBER LOURMART CN-15-LC, 230 V- 50/60 Hz).

R_f_ (Retention factor) was calculated using the following formula [11]:


$$Rf=\frac{DistanceTravelledbySolute}{DistanceTravelledbySolvent}$$

#### Headspace analysis of GC-MS.

Hexane, dichloromethane, and ethyl acetate extracts of *in vitro* plantlets and agarwood of *G. walla* were used for the headspace analysis of GC-MS to detect volatile compounds and their comparison. GC-MS analysis was performed on 5975C gas chromatograph (Agilent technologies, USA, www.agilent.com) fitted with a fused silica HP-5MS capillary column (30 m ×  0.25 mm; film thickness 0.25 μm). Helium was used as carrier gas at a flow rate of 2 mL/min. The gas chromatograph was coupled with 7693 mass selective detector (Agilent technologies, USA, www.agilent.com). Headspace auto-samplers were used to have provision to pressurize the vial at a constant pressure equivalent to the column inlet pressure for better quantification. Vials were placed on auto-sampler and generated results for each sample was obtained as chromatograms showing the chemicals composition with the help of the software attached to the instrument.

## Results

### Establishment of shoot tip and axillary bud cultures

**Fig 1:**
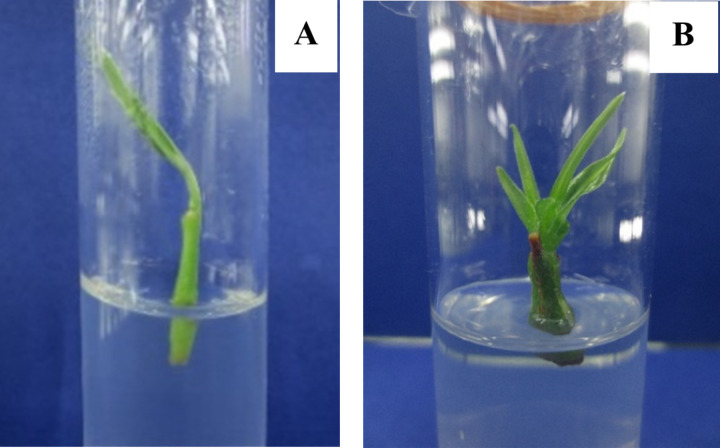
Effect of explants on establishment of cultures on MS media supplemented with 1.0 mg/L BAP. (A) shoot tip cultures. (B) axillary bud cultures.

Both shoot tips and axillary buds were successfully established on MS medium supplemented with 1.0 mg/L of BAP (Fig 1).

**Fig 2:**
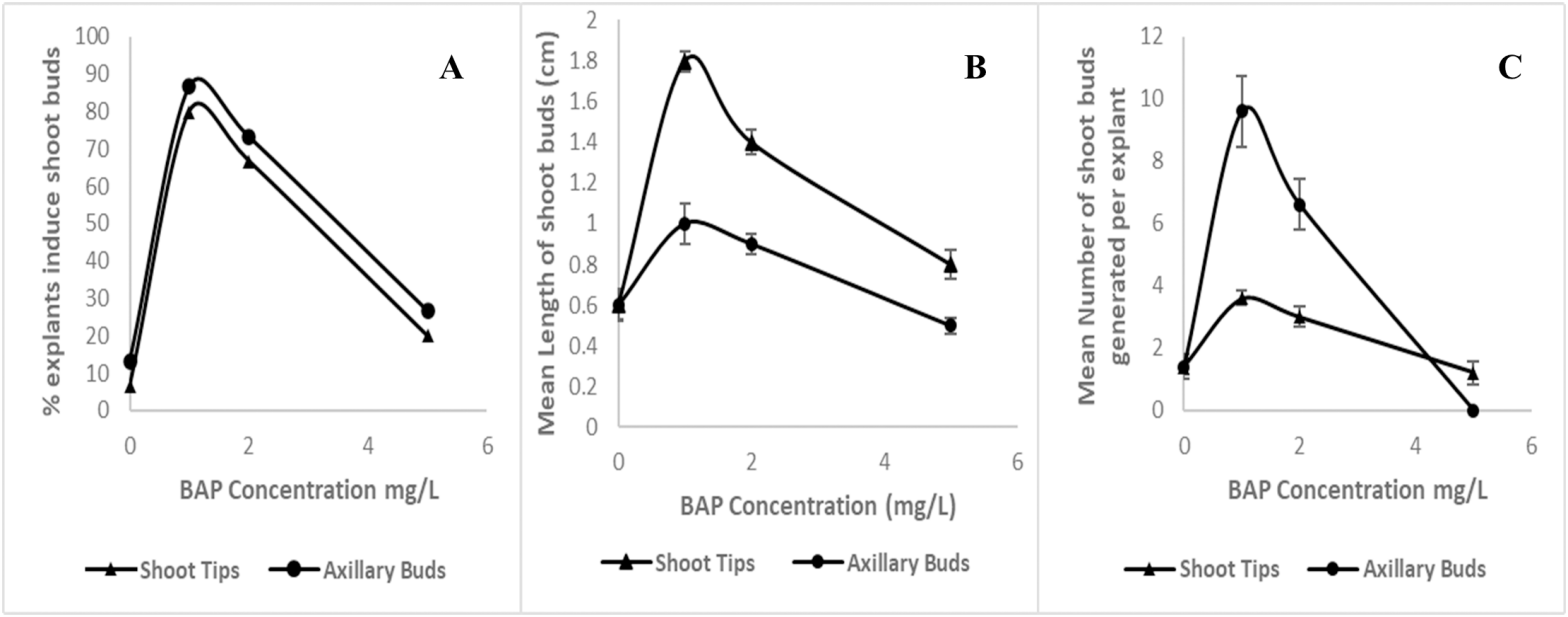
Effect of BAP concentrations in MS media on shoot induction from shoot tip and axillary bud explants. (A) percentage explants induced shoot buds out of 25 shoots. (B) mean length (cm) of shoot buds eight weeks after inoculation (2^nd^ subculture). (C) mean number of shoot buds 16 weeks after inoculation (4^th^ subculture).

**Fig 3:**
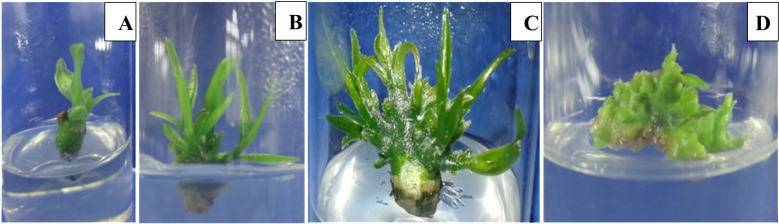
Growing axillary bud cultures (at 16 weeks, at the end of 4^th^ subculture) on MS media supplemented with different concentrations of BAP. (A) 0 BAP. (B) 1.0 mg/L BAP.(C) 2.0 mg/L BAP.(D) 5.0 mg/L BAP.

MS medium supplemented with 1.0 mg/L BAP was found to be best for inducing shoot buds from both shoot tips (80.0%) and axillary buds (86.7%) (Fig 2A). The same medium was proven to be superior by significantly increasing the mean length of regenerated shoot buds (1.8 cm and 1.0 cm for shoot tips and axillary buds, respectively) after eight weeks of inoculation (Fig 2B). After the 16th week of inoculation (4^th^ subculture), the highest number of shoot buds per explant (3.6 and 9.6 for shoot tips and axillary buds, respectively) was regenerated in the same medium (Fig 2C). MS media, without any PGRs, was not efficient in inducing the shoot buds from both explants (6.7% and 13.3%) (Fig 2A). Axillary bud explants, which were in MS media containing 5.0 mg/L BAP, started to proliferate rapidly, but when they were subsequently transferred to the same medium, the shoot buds were twisty and further elongation was not recorded (Fig 2B and Fig 3). Multiplied shoots on medium consisted of 5.0 mg/L BAP became vitrified and showed abnormalities and could not be isolated for further multiplication. Based on the results of this experiment, MS medium supplemented with 1.0 and 2.0 mg/L BAP were selected for the elongation and further multiplication of proliferated shoots in the second experiment.

### Elongation and further multiplication of *in vitro* established shoots

In the first experiment, both shoot tip and axillary bud explants established on MS medium supplemented with 30 g/L sucrose and 1.0 or 2.0 mg/L BAP did not elongate beyond 2 cm (Fig 2A). Selection of concentration and combination of PGRs is critical for shoot multiplication and elongation before rooting and acclimatization of micropropagated shoots. Thus, it was decided to investigate the effect of higher concentration (40 g/L) of sucrose in combination with 0, 1.0 or 2.0 mg/L BAP or 0. 1 mg/L GA_3_ on shoot elongation and further multiplication. When the shoots were sub-cultured continuously on to the hormone-free MS medium, irrespective of the sugar concentration (30 or 40 g/L), yellowing of leaves, ultimately necrosis and senescence was observed. Shoots transferred to MS media supplemented with 0.1 mg/L GA_3_ also showed stunted stem growth with elongated leaves disproportional to the stem size. Eventually, chlorosis and shedding leaves followed by senescence of shoots occurred in this medium. Furthermore, none of the shoots transferred to the media containing 1.0 or 2.0 mg/L BAP alone did not elongate beyond 2.0 cm, both at 30 and 40 mg/L sucrose concentration (Table 1). However, shoots grown on medium containing 1.0 mg/L BAP showed significantly higher elongation and multiplication compared to medium consisted with 2.0 mg/L BAP (Table 1). Furthermore, lower concentrations of IBA (0.1 mg/L) was incorporated into MS medium supplemented only with 1.0 mg/L BAP to investigate the combined effect of BAP and IBA on shoot elongation and multiplication.

**Fig 4:**
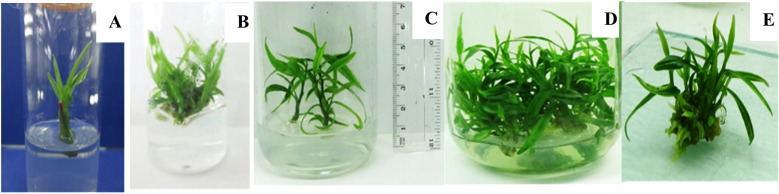
Elongation and multiplication of shoots on MS media supplemented with 40 g/L of sucrose,1.0 mg/L BAP and 0.1 mg/L IBA. (A) at the time of transferring (16^th^ week of establishment). (B) at the end of 1^st^ subculture (20^th^ week of establishment). (C) at the beginning of 2^nd^ subculture (shoots cluster separated). (D) multiplication of shoots at the end of 2^nd^ subculture (at the end of 24^th^ week of establishment). (E)one cluster of multiplied shoots at the end of 24^th^ week.

Interestingly, significantly higher elongation (4.0 cm) and multiplication (10.6 shoots per cluster) of shoots could be achieved eight weeks after transferring the shoots (24^th^ weeks after establishing) to MS medium supplemented with 1.0 mg/L BAP, 0.1 mg/L IBA and 40 g/L sucrose (Table 1, Fig 4). Furthermore, when 40 g/L of sucrose was added to full MS medium supplemented with 1.0 mg/L BAP and 0.1 mg/L IBA performed well compared to 30 g/L of sucrose with same concentrations of BAP and IBA showing that there is an interactive effect (*P* ≤ 0.05) between sucrose concentration when 1.0 mg/L BAP was in combination with 0.1 mg/L IBA.

### *In vitro* rooting of *G. walla* on MS medium

**Fig 5:**
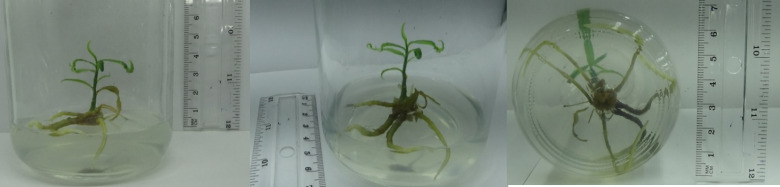
*In vitro* rooted plantlets in½  MS media supplemented with 40 g/L sucrose and 1.0 mg/L IAA.

Adventitious roots initiation was observed after five weeks of transferring the shoots into the rooting media. Highest rooting percentage (66.7 ± 5.27%) with highest mean root length (6.15 ± 0.34 cm) was observed when the microshoots were transferred to ½  MS medium fortified with 1.0 mg/L IAA and 40 g/L sucrose concentration. However, this treatment produced lowest mean number of roots/shoots (1.56 ± 0.241). The highest average number of roots was observed in ½  MS medium supplemented with 0.5 mg/L IAA either with 40 (2.45 ± 0.47) or 30 g/L (2.31 ±  0.24) sucrose concentrations (Table 2 and Fig 5).

**Table 2:** Effect of ½  MS media supplemented with different concentration of sucrose and IAA in root induction.

½ MS media		Percentage of rooted shoots (%)	Number of roots/shoot	Root length (cm)
Sucrose (g/L)	Concentrations of IAA (mg/l)			
30	0.5	41.7 ± 4.08b	2.31 ± 0.24a	5.10 ± 0.26b
30	1.0	33.3 ± 5.27b	2.10 ± 0.46a	0.60 ± 0.01d
40	0.5	33.3 ± 7.45b	2.45 ± 0.47a	0.97 ± 0.14c
40	1.0	66.7 ± 5.27a	1.56 ± 0.24b	6.15 ± 0.34a

Data represent mean ±  SE. Data followed by the different letters in a column are significant at *P* ≤ 0.05.

Moreover, among all treatments, ½  MS media supplemented with 40 g/L of sucrose, induced roots well, compared to 30 g/L of sucrose fortified ½  MS media. However, all the treatments produced very fragile looking roots initiated from the callus produced at the base of the shoots (Fig 5).

### *In vitro* rooting of *G. walla* on sand medium

Root initiation was observed 5–6 weeks after transferring the shoots into the sand medium. The highest percentage (93.3 ± 4.08%) of rooting was recorded, when micro-shoots were transferred to sand without IAA or IBA treatment. Eight weeks after transferring, the lowest percentage of rooted shoots (33.3 ± 5.27%) were recorded when micro-shoots were dipped in 1000 mg/L IBA for 30 minutes before planting in the sand bottle (Table 3). Majority of the shoots treated with 1000 mg/L IBA became yellow and die back was observed as in the preliminary study conducted under *in vitro* conditions [7].

**Table 3:** Effect of pulsing treatments of shoots using 1000 ppm IAA and IBA for different pulsing period on *ex vitro* rooting.

Type of auxin	Pulsing period (min)	Percentage of rooted shoots (%)	Number of roots per shoot	Root length per shoot (cm)
1000 mg/L IAA	15	66.6 ± 5.27b	1.83 ± 0.16b	1.69 ± 0.19a
1000 mg/L IAA	30	73.3 ± 8.50b	2.21 ± 0.21b	1.79 ± 0.17a
1000 mg/L IBA	15	46.7 ± 3.33c	**4.00 ± 0.61a**	1.41 ± 0.17a
1000 mg/L IBA	30	33.3 ± 5.27c	**3.88 ± 0.55a**	1.59 ± 0.17a
Control	15	**93.3 ± 4.08a**	2.21 ± 0.32b	1.75 ± 0.30a
Control	30	**93.3 ± 4.08a**	2.20 ± 0.24b	1.75 ± 0.30a

Data represent mean ±  SE. Data followed by the different letter within each column are significant at *P* ≤ 0.05.

Statistical analysis showed that there was a significant difference in inducing number of roots per shoot in between IAA and IBA containing media (Table 3). The highest average number of roots per shoot was observed in the shoots, pulsed with 1000 mg/L IBA for 15 minutes (4.0), followed by 1000 mg/L IBA for 30 minutes (3.88).

**Fig 6:**
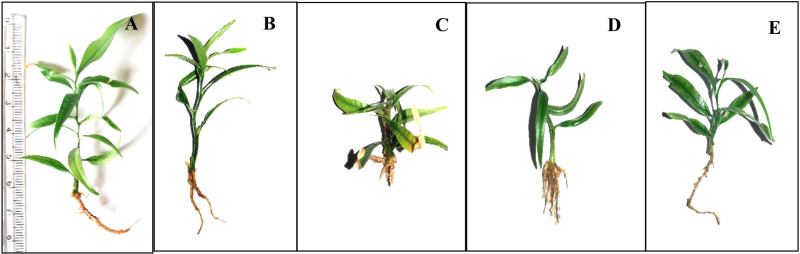
Rooted shoots on sand medium with different treatments. (A) 1000 mg/L IAA for 15 minutes. (B) 1000 mg/L IAA for 30 minutes. (C) 1000 mg/L IBA for 15 minutes. (D) 1000 mg/L IBA for 30 minutes.(E) control.

Even though, there was no significant difference among treatments for the average root length per shoot at *P* ≤ 0.05 probability level (Table 3), the highest root length (1.79 cm) was observed when the shoots treated with 1000 mg/L IAA for 30 minutes. Meanwhile, shortest root (1.41 cm) was recorded under 1000 mg/L IBA for 15 minutes treatment (Fig 6).

Furthermore, development of good quality roots without callus was observed in the sand medium irrespective of the hormones used for rooting and pulsing time.

### Acclimatization

**Fig 7:**
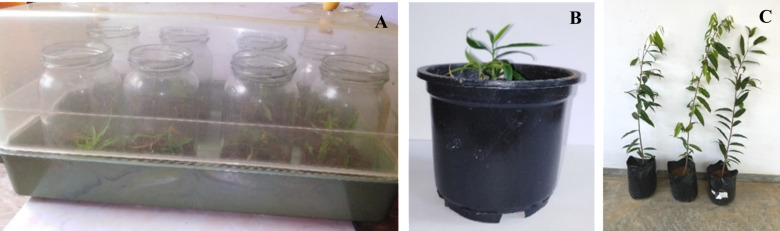
Plants rooted on sand medium at different stages of growth. (A and B) Plants during acclimatization. (C) *in vitro* propagated one year old *G. walla* plants.

All the rooted shoots, produced in sand medium were successfully acclimatized within four weeks and 83.6% of them were grown to full plants in the sand: soil: compost media at 1:1:1 ratio (Fig 7). However, none of the plants rooted on MS media survived during acclimatization showing the superiority of the sand medium for rooting of micropropagated *G. walla* plants.

### Comparison of chemical constituents present in micropropagated plantlets and agarwood of *G. walla* using TLC and GC-MS

#### TLC fingerprints of *in vitro* plantlets and agarwood.

**Fig 8:**
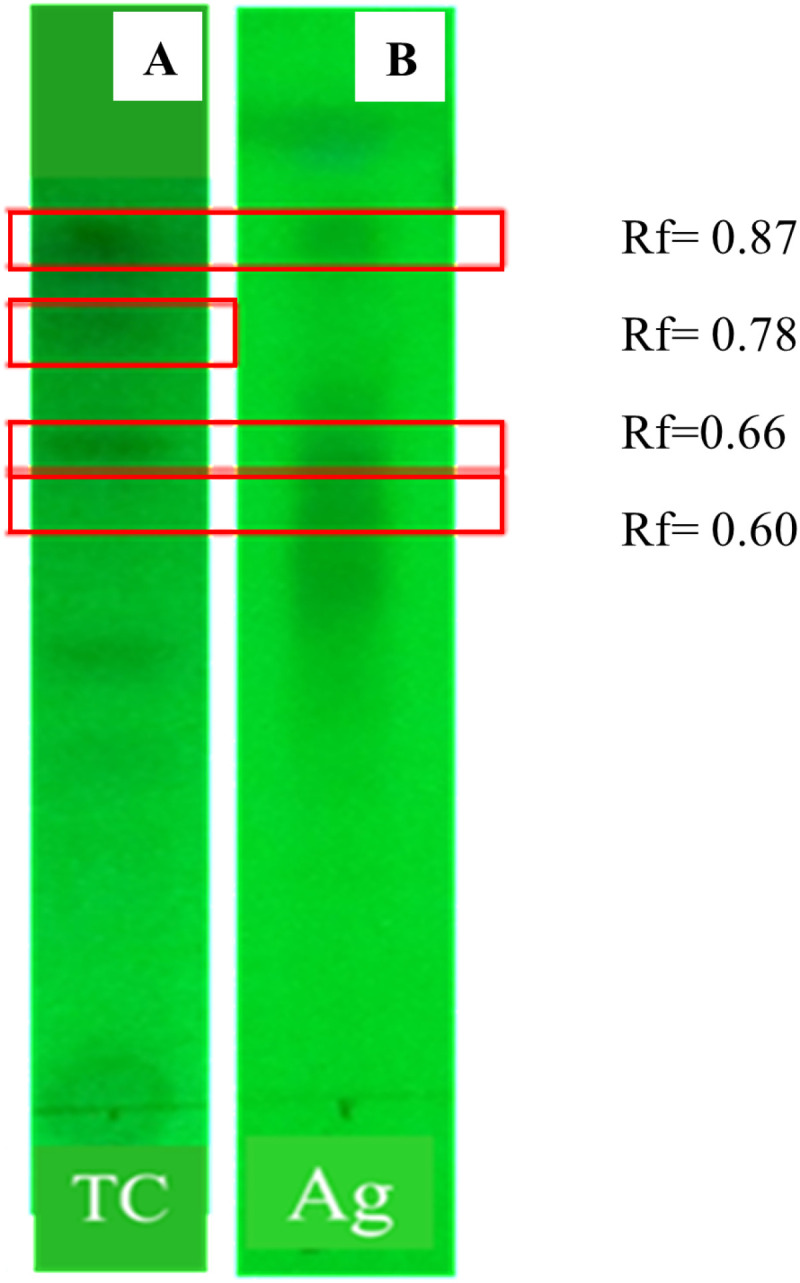
TLC fingerprints developed for comparing *in vitro* grown plantlets with agarwood of *G. walla* under 15% methanol: 85% chloroform solvent system (v/v). (A) *in vitro* plantlets (TC). (B)agarwood of *G. walla* (Ag).

Ethyl acetate extracts of *in vitro* grown plantlets and agarwood produced similar spots at the retention factors of 0.60, 0.66 and 0.87 (Fig 8) indicating that three similar, less polar compounds might be present in *in vitro* grown plantlets and agarwood of *G. walla,* under 15% methanol: 85% chloroform solvent (v/v) system.

#### Gas chromatography and mass spectrometric profiles of *in vitro* plantlets and agarwood.

In the GC-MS profile of micropropagated plantlets, total of 46 compounds were identified while agarwood sample of *G. walla* contained total of 95 compounds. Hexane, dichloromethane and ethyl acetate extracts of micropropagated shoots contained 7, 7 and 32 compounds respectively, while agarwood extracts contained 63, 15 and 17 compounds respectively. All the seven compounds detected in hexane extracts of micropropagated shoots were eluted before 16.13 (RI) while all the chemicals except 4 Hydroxypyridine 1 –oxide present in hexane extracts of agarwood eluted after 16.13 minutes. Furthermore, 4 Hydroxypyridine 1 –oxide, which was eluted at 15.27 minutes was common for both agarwood and hexane extracts (Supplementary information).

In the present study, many compounds identified in the agarwood GC-MS profile of *G. walla* were already reported in earlier studies done in agarwood producing tree species, especially in *Aquillaria* species. Agarospirol (0.5%) [12–14], aristolene (6.5%) [14,15], β -neoclovene (4.5%) [16], heptadecane, 2-methyl (1.0%) [14] and octadecane, 1-chloro (2.0%) 14] were the compounds identified in hexane extracts of agarwood (Supplementary Information). Furthermore, nonadecane (2.3%), aristolene (7.1%) [14], hexadecane (5.0%) [14], eicosane (4.1%) [15] and octacosane (5.0%) [17] were the compounds identified in dichloromethane extract of agarwood. However, none of these chemicals were present in hexane and dichloromethane extracts of microshoots. Interestingly, 2-tetradecene (11.9%) [18] and 1-hexadecene (23.5%) [14], E-15-Heptadecenal (19.8%) and 18-Norabietane (0.6%) were detected in ethyl acetate extracts of both agarwood and micro-shoots as reported in the earlier studies for *Aquillaria* species.

Compounds identified in the micropropagated plantlets such as, dodecane [14], 2,6,10-trimethyl-, 12-octadecenoic acidmethyl ester [14], hexanedioic acid, mono(2-ethylhexyl) ester [14], 2-tetradecene [18], 1-hexadecene [14], E-15-heptadecenal, 1-octadecene [15,19], eicosane [19] and heptadecane [19] were also reported in previous studies done in development of chemical profiles of *Aquillaria* species (Supplementary information).

## Discussion

### Development of a micropropgataion procedure for mass cultivation of *G. walla*

The present study was focused on direct proliferation of the shoot tips and axillary bud explants of *G. walla* since it is the most reliable method for clonal propagation to achieve the genetic uniformity among the progenies. Even though, proliferated shoot tip explants were taller than the shoots proliferated from the axillary buds, the mean number of generated shoot buds during establishment stage was higher in the axillary bud explants (Fig 2C). Furthermore, the availability of axillary buds from a single mother plant is higher than the shoot tips. Thus, axillary bud explants can be considered as a better source of explants for the establishment of *G. walla* shoot cultures.

In agreement with the results of earlier studies [20], low concentrations of BAP was effective in stimulating shoot buds initiation from both shoot tips and axillary buds. The increase of BAP concentration above 1.0 mg/L BAP showed drastic reduction in shoot establishment as well as shoot elongation. At highest BAP concentration (5.0 mg/L) further elongation of initiated shoot buds did not occur. Moreover, those shoots showed symptoms of vitrification which may happen as a result of high cell division associated with high concentrations of cytokinins [21] and inhibition of adventitious meristem elongation [20,22].

Micropropagated plantlets growing under *in vitro* conditions are not autotrophic and therefore need to have a supply of energy source for *in vitro* cell growth, maintenance, and differentiation [23]. Previous studies stated that sugar alone or through its interaction with different phytohormones and nitrogen can induce or suppress many growth-related gene responses in higher plants [24,25]. Thus, in the present study importance of maintaining the balance between the carbon source and PGRs for the shoot elongation and multiplication during *in vitro* growth of *G. walla* was investigated. The shoots transferred to MS medium without BAP had low multiplication, slow rate of shoot elongation, and ultimately shoots became necrotic and died irrespective of the sugar concentration of the medium. In agreement with the results of the present study differences in the shoot growth, shoot tip dieback, defoliation, and necrosis has been reported when nodal explants of avocado were grown on hormone-free media for 30 days [26]. Furthermore, MS media supplemented only with BAP (1.0 or 2.0 mg/L) also did not elongate at higher sucrose concentrations (40 g/L) than the recommended level (30 g/L). Even though, gibberellic acid (GA_3_) cause internodal elongation [21], in the present study further elongation of microshoots could not be achieved on MS media supplemented with 0.1 mg/L GA_3_ both at 30 and 40 g/L sucrose concentrations. In contrast, stunted stem growth occurred while elongating the leaves disproportional to the size of the stem. Eventually, chlorosis and leaf shedding leading to senescence of shoots was observed. In agreement with the results of the present study, deleterious effects of GA_3_ on elongation and rooting of *Prunus instititia* L. rootstock [27] resulting chlorosis and apical die-back of *in vitro* shoots has been reported [27]. Furthermore, GA_3_ alone in the media had no effect on multiplication and elongation of tea shoots [21]. Interestingly, shoots transferred to MS medium supplemented with 1 mg/L BAP combined with 0.1 mg/L IBA performed well at both sucrose concentration and better performance was observed at 40 g/L sucrose concentration compared to 30 g/L concentration (Table 1). Thus, it may be possible that 30 g/L sucrose concentration is insufficient to supply the energy required for the high cell division and differentiation occurred in the medium supplemented with 1.0 mg/L BAP combined with 0.1 mg/L IBA while increase of the sucrose concentration up to 40 g/L may supply the required level of energy for the growth and development. Furthermore, the exogenous supply of sucrose may increase the endogenous content of carbohydrate stocks in *in vitro* plants, which may accelerate physiological adaptations of the *in vitro* shoots [28]. In addition, higher sucrose concentration of the *in vitro* medium may support water conservation, maintain the osmotic potential of cells by acting as an osmoticum and consequently reducing vitrification of *in vitro* cultures [28]. In agreement with the results of the present study 40 g/L of sucrose has been shown to increase the number of shoots/ explant and shoot length of developed shoots in *Harpagophytum procumbens* [29] and taller shoots in *in vitro* beech cultures with lower rate of vitrification [30]. In the present study, half strength of MS medium was selected for *in vitro* root induction, since the rooting ability of *in vitro* shoots have been reported to be affected by the mineral concentration of the medium [31]. In many species such as *Dendrocalamus longispathus* [32], *Chlorophytum borivilianum* [33] and *Dalbergia sissoo* [34], the percentage of rooted shoots was higher when shoots were rooted on half strength MS medium.

A higher concentration of sucrose (40 g/L) than the recommendation (30 g/L) in MS medium with reduced nitrogen strength by half, showed better *in vitro* root induction in the current study. In agreement with the results of the present study, sucrose at concentrations of 40–50 g/L is reported to increase the number of roots (5.8–6.0) in the media containing reduced level of nitrogenous salts in *Helleborus niger* microplants [35]. The rooting ability is strongly dependent on the carbon to nitrogen ratio of plants [36]. The effect of modification in carbon abundance has an impact on nitrogen metabolism and *vice versa*. An integration of C/N nutrient signals with the phytohormone act on developmental process in plants [37]. Thus, the presence of higher sucrose concentration in the culture medium may contributed to the process of cell division in the root apical meristem, which caused an increase in root length [38].

Auxins play an important role in root formation. The composition of rooting media, concentration, and type of auxin has been shown to effect the *in vitro* rooting [39]. Many studies have shown that exogenous application of auxins results in increased initiation of lateral roots. Thus, series of experiments were conducted to develop a protocol for rooting in *Gyrinops walla* microshoots. Results of preliminary study [7] showed that percentage rooting of microshoots transferred to ½  MS medium containing both NAA and IBA was very less ( < 50%). At higher IBA and NAA concentration (2.0 mg/L) none of the shoots produced roots while highest rooting percentage for IBA (25%) was achieved when media were supplemented with 0.1 or 0.5 mg/L IBA with 40 g/L sucrose. Most of the shoots cultured on media containing IBA and NAA started chlorosis and senescence indicating that continuous exposure to auxin rich medium does not favour the growth of micro shoots [7].

IAA is one of the effective auxins used in *in vitro* rooting of shoots in many plant species. In agreement with the results of the present study *in vitro* root induction and growth was highly inhibited with NAA and IBA than with IAA in apple variety ‘Jork 9’ [40].

*Ex vitro* rooting of micro shoots is an efficient technique in many of the difficult to root woody plant species [41,42]. *Ex vitro* rooting was found superior since plantlets developed through this method had lateral roots without any callus at the base of microcuttings, higher root lengths, rooting rates, and transplant survival rate compared to the *in vitro* rooted plantlets [43]. In the present study, rooting experiment on sand medium was conducted to improve the success rate of rooting of micro shoots as well as to develop a new technique to overcome drawbacks occurred in the *in vitro* rooting experiments.

Tissue-cultured microshoots of 23 tree species and bamboo had developed roots in the sand and these microplants can easily survive in the field because of their healthy roots grown in the sand [44]. Therefore, sand was selected as the rooting medium replacing the MS medium in the present study.

Our study showed the possibilities of successful rooting of *G. walla* without any auxin treatments on sand medium in agreement with the results obtained during *ex vitro* rooting of two *Lonicera. caerulea* var. kamtschatica cultivars ‘Wojtek’ and ‘Zojka’ [45].

On the other hand, lowest rooting percentage was observed, when the shoots were treated with IBA. In contrast, IBA has been reported as most effective in inducing roots in *Siraitia grosvenorii* [46]. Toxicity at high IBA concentration might be the reason for reduction in the survival and root initiation observed in the present study. In many research, lower concentration of IBA for shorter period of treatment were reported as highly efficient in inducing roots under the *ex vitro* condition. *Ex vitro* rooting of micropropagated shoots of *Bauhinia racemosa* Lam. treated with IBA 400 mg/L for seven minutes [47] and *Couroupita guiane*ns, treated with 400 mg/L of IBA for five minutes [48] had shown effective for rooting. It is also confirmed that *Solanum muricatum* Aiton. cuttings treated with 500 mg/L IBA were more effective than the concentration of 1000 mg/L [49]. In the present study higher efficacy of rooting on sand medium was observed when the *in vitro* grown shoots were treated with IAA compared with IBA for 15 minutes and 30 minutes. The photooxidation of IAA occurs rapidly (50% within 24 hours) compared to that of IBA (10% within 24 hours [50]. Thus, IAA may also not effective in rooting of *G. walla* microshoots due to this character. This implies that internal auxin concentrations of the micro-shoots are adequate for root initiation as observed in the untreated micro-shoots.

Furthermore, all the roots developed in the sand medium were healthy and did not form callus at root base. Under the *in vitro* rooting conditions, the roots were developed from the callus base, which were fragile and difficult to acclimatize. *In vitro* media are rich in mineral nutrients, plant growth regulators as well as carbohydrates. Thus, cells at base of shoots can absorb nutrients easily by diffusion resulting high rate of cell division and multiplication compared to the microshoots growing on sand media with low mineral nutrient contents resulting callus formation.

Furthermore, sand media do not supply carbohydrate to the microshoots and become autotrophic during rooting process resulting better growth with morphologically strong and well-developed root system that sustains under field conditions [43].

Acclimatization is a critical stage of micropropagation of woody species. Commercialization of micropropagation technology has been hampered by the poor rate of survival of *in vitro* raised plantlets during acclimatization [28,51]. Many *in vitro* propagated plant species show a high mortality rate during the process of transferring to *ex vitro* conditions [52].

In the present study, 83.6% survival rate was recorded during acclimatization of plants rooted on sand medium while none of the plants rooted on MS medium could be acclimatized. This results implies the importance of successful rooting [53] for acclimatization and field establishment of plantlets.

#### Comparison of chemical constituents present in *in vitro* plantlets and agarwood of *G. walla* by TLC and GC-MS.

TLC fingerprint with a visible pattern of bands provides fundamental data which can be used to determine the number of components in a mixture and to identity them by comparing with *Rf* of a known compound when both run on the same TLC plate using the same eluent [54]. In the present study, similar spots at the retention factors of 0.60, 0.66, and 0.87 of ethyl acetate extracts of tissue culture plantlets and agarwood indicate that *in vitro* grown plantlets also produce very similar compounds to chemicals produced by agarwood of *G. walla*. Thus, it may be possible to use these bands as TLC fingerprints for identifying plant samples derived from *G. walla*.

Plant cells can be considered as miniature factories successfully producing high-value secondary metabolites [55]. Previous studies done in several plant species proved the potential for the industrial production of secondary metabolites through plant cell cultures, such as berberine [56], rosmarinic acid [57] and sanguinarine [58]. Compounds identified in the GC-MS belonged to the groups of aldehydes, phenol, ether, ketone, sesquiterpenes hydrocarbons, oxygenated sesquiterpenes, fatty acid methyl esters, and sterols.

Interestingly GC-MS results obtained by hexane, dichloromethane and ethyl acetate extracts of microshoots and agarwood confirmed the results reported for TLC finger prints of *G. walla* callus extracts [10], where phytochemicals present in callus could be visualized clearly by using thin layer chromatography when calli were extracted with ethyl acetate compared to hexane and dichloromethane extracts. Furthermore, TLC profiles obtained in the present study produced three similar compounds based on retention time while GC-MS results confirmed it by resulting five similar compounds in ethyl acetate extracts of both agarwood and microshoots.

Most of the compounds detected in the present study have not been reported elsewhere and some of the important compounds identified in this study were already reported in earlier studies done for agarwood of *Aquillaria* species. However, the compounds such as Agarospirol, aristolene and β –neoclovene which contribute to fragrance were not present in the microshoots. In the present study, microshoots were grown in nutrient rich environment and it is a known fact that agarwood resinous compounds are produced as a response to fungal invasions. Thus, it may be possible to enhance the production of resinous compounds through the *in vitro* shoot cultures of *G. walla* by using elicitors such as salicylic acid, chitosan and methyl jasmonate.

## Conclusions

Shoot tips (80.0%) and axillary buds (86.0%) could successfully be established on MS medium supplemented with 1.0 mg/L BAP. Further multiplication (10.6 shoot buds/ regenerated shoot) and elongation (4.0 cm) of established shoot buds could be achieved by transferring to MS medium supplemented with 40 g/L sucrose and 1.0 mg/L BAP combined with 0.1 mg/L IBA. None of the shoots rooted on *in vitro* MS media containing IAA, IBA or NAA could be acclimatized. Higher rooting efficiency (93.3%) could be achieved by transferring microshoots to sand medium without application of auxins and 83.6% of them were successfully acclimatized in the medium containing sand, soil, and compost at 1:1:1 ratio.

TLC fingerprints of *in vitro* plantlets and agarwood revealed the presence of three similar compounds at *R*_*f*_ values of 0.60, 0.66 and 0.87, which could be used as possible markers for *G. walla*. In the GC-MS analysis, 4-Hydroxypyridine 1-oxide, 2-tetradecene (23.2%), 1-hexadecene (0.3%), E-15-heptadecenal (19.8%), 18-norabietane (0.6%) and eicosane (0.4%) were the similar compounds identified in both *in vitro* plantlets and agarwood of *G.walla.*

The present study successfully standardized the culture conditions of each stage of micropropagation of *G. walla* and elucidates the possibilities of producing agarwood resinous compounds using the *in vitro* shoot culture techniques.

### Key message

Efficient micropropagation protocol was developed for rapid regeneration and multiplication of *G. walla,* an endangered species, to conserve from extinction. TLC and GC-MS chemical profiles of *in vitro* plantlets and agarwood extracts were compared, and similar resinous compounds were identified.

## Supporting information

S1 Fig GC-MS Chromatograms of different solvent extracts of *in vitro* propagated plantlets and agarwood of *G. walla* . (A) Hexane extract, (B) dichloromethane extract, ethyl acetate extract of *in vitro* propagated plantlets and (D) hexane extract (E), dichloromethane extract (F), ethyl acetate extract of agarwood of *G.walla.
* (PDF)

S1 Table Effect of BAP concentrations in MS media on percentage explants induced shoot buds, mean length of shoot bud and mean no. of shoot buds from shoot tip and axillary bud explants. (PDF)

S2 Table Comparison of phyto-chemicals identified by GC-MS analysis in agarwood and microshoots of *G. walla.
* (PDF)

## Abbreviations

TLC: Thin Layer Chromatography
GC-MS: Gas chromatography- mass spectrometry
PGR: Plant growth regulator
BAP: 6-Benzylaminopurine
NAA: 1-Naphthaleneacetic acid
IBA: Indole-3-butyric acid
IAA: Indole-3-acetic acid
MS: Murashige and Skoog medium
