# Supplementary material for: Rapid micropropagation and chemical profiling of in vitro plantlets and agarwood of Gyrinops walla Gaertn. by gas-chromatography and mass-spectrometry
Source: PLoS One. 2025 Apr 8;20(4):e0321049. doi: 10.1371/journal.pone.0321049 (PMC11978006; doi:10.1371/journal.pone.0321049)
Supplement: S1 Fig — (PDF) [file pone.0321049.s001.pdf]

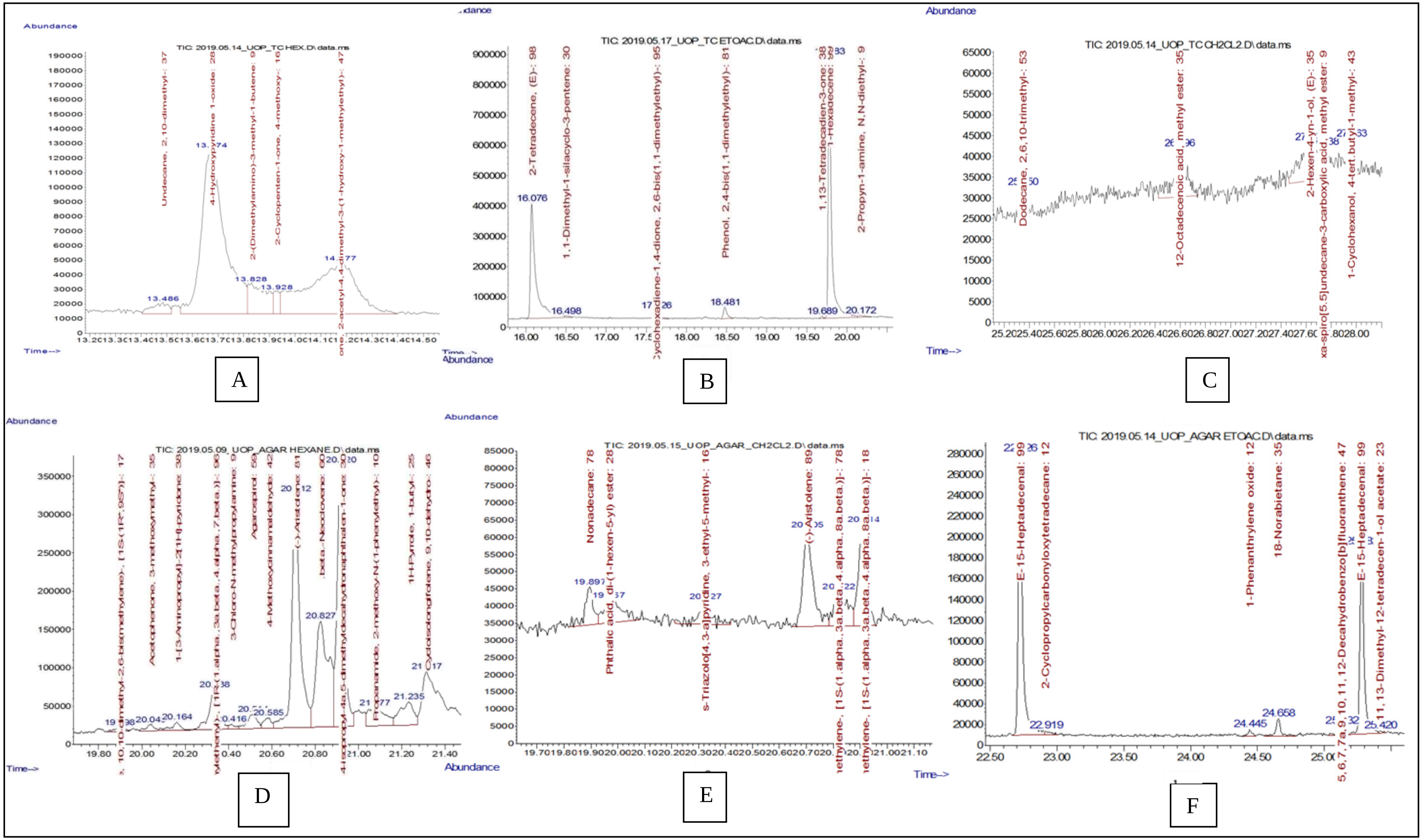

S1 Fig. GC-MS Chromatograms of different solvent extracts of *in vitro* propagated plantlets and agarwood of *G. walla*

(A) hexane extract, (B) dichloromethane extract, (C) ethyl acetate extract of *in vitro* propagated plantlets and (D) hexane extract, (E) dichloromethane extract, (F) ethyl acetate extract of *agarwood of G.walla*
