# Supplementary material for: Rapid micropropagation and chemical profiling of in vitro plantlets and agarwood of Gyrinops walla Gaertn. by gas-chromatography and mass-spectrometry
Source: PLoS One. 2025 Apr 8;20(4):e0321049. doi: 10.1371/journal.pone.0321049 (PMC11978006; doi:10.1371/journal.pone.0321049)
Supplement: S1 Table — (PDF) [file pone.0321049.s002.pdf]

S1 Table. Effect of BAP concentrations in MS media on percentage explants induced shoot buds, mean length of shoot bud and mean no. of shoot buds from shoot tip and axillary bud explants

Explants	MS media with different concentrations of BAP (mg/L)	% of explants induced shoot buds after 8 weeks of inoculation	Mean length (cm) of shoot bud after 8 weeks of inoculation	Mean no. of shoot buds generated per explants after 16 weeks of inoculation
Shoot tips	0	6.7	0.6±0.07c	1.4±0.24b
	1	80.0	1.8±0.05a	3.6±0.24a
	2	66.7	1.4±0.06b	3.0±0.32a
	5	20.0	0.8±0.07c	1.2±0.37b
Axillary buds	0	13.3	0.6±0.08bc	1.4±0.40c
	1	86.7	1.0±0.10a	9.6±1.12a
	2	73.3	0.9±0.05ab	6.6±0.81b
	5	26.7	0.5±0.04c	0c
