# Supplementary material for: Rapid micropropagation and chemical profiling of in vitro plantlets and agarwood of Gyrinops walla Gaertn. by gas-chromatography and mass-spectrometry
Source: PLoS One. 2025 Apr 8;20(4):e0321049. doi: 10.1371/journal.pone.0321049 (PMC11978006; doi:10.1371/journal.pone.0321049)
Supplement: S2 Table — (PDF) [file pone.0321049.s003.pdf]

S2 Table. **Comparison of phyto-chemicals identified by GC-MS analysis in agarwood and microshoots of *G. walla***

**Retention Indices**	**Agarwood**	**Micro Shoots**
**Hexane Extracts**		
13.486		Undecane, 2, 10-dimethyl-
13.674		4-Hydroxypyridine 1-oxide
13.828		2-(Dimethylamino)-3-methyl-1butene
13.928		2-Cyclopenten-1-one, 4-methoxy
14.177		Cyclopentanone, 2-acetyl-4,4-dimethyl-3-(1-hydroxy-1-methylethyl)
**15.266**		**4-Hydroxypyridine 1-oxide**
**15.408**	**4-Hydroxypyridine 1-oxide**	
16.134		1-iodo-2-methylundecane
16.875	Benzene, 1-ethenyl-3,5-dimethyl	
17.229	Naphthalene, 1,2,4a,5,8,8a-hexahydro-4,7-dimethyl-1-(1-methylethyl)-, (1.alpha.,4a.beta.,8a.alpha.)-(.+-.)	
17.362	Bicyclo [7.2.0]undeo-4-ene. 4.11.11-trimethyl-8-methylene	
18.295	1,2,5-Oxadiazol-3-amino, 4-(3-methoxyphenoxy)	
18.394	1-7-(1-methylethenyl)-. [1S-(1. alpha., 7.alpha., 8a. beta.)]	
18.481	Phenol. 2.4-bis(1.1-dimethylethyl)	
18.632	Pyridine. 1,2,3,6-tetrahydro-4-phenyl	
19.113	-. [ar-(1a.alpha., 4a.alpha.,7.beta.,7abeta.,7b.alpha.)]	

19.898	10,10-dimethyl-2,6-bis(methylene)-,[1S-(1R*, 9S*)]	
**20.514**	**Agarospirol** [9-11]	
**20.712**	**Aristolene** [11, 12]	
**20.827**	**β –Neoclovene** [13]	
20.920	4-Isopropyl-4a,5-dimethyldecahydronaphthalen-1one	
21.077	Propanamide, 2-methoxy-N-(1-phenylethyl)	
21.235	1H-Pyrrole, 1butyl	
21.317	Cycloisolongifolene, 9,10-dehydro	
21.581	Sulfurous acid, nonyl 2-propyl ester	
21.679	Butyric acid, 3-methyl-3-[2-isopropylphenyl)	
21.780	2,2-Dimethyl-4-hydroxy-7-methoxy-chroman	
21.871	a-tetramethyl-,(1a.alpha.,7.alpha.,7b.alpha.)	
21.939	Cyclobutane, tetrakis(1-methylethylldene)	
22.049	Pyridine, 2,4-dimethyl	
22.114	Triquinacene, 1,4,7-tris(methoxy)	
22.199	Dimethylthexylsiiyi chloride	
22.250	5,6-Dihydro-2-(4tolyl)-4H-1,3-oxazin-5-one	
22.527	m-Menth-1(7)-ene, (R)-(-)	
22.745	Cyclopenta[c]pyran-7-carboxaldehyde, 4-[(acetyloxy)methyl]	
22.956	Cyclohexanecarbonitrile, 1-(1-piperidinyl)	
23.116	1,3,4-Oxadiazole, 2-(4-dimethylaminophenyl)	
23.412	1,8-Nonadien-3-yne, 2.8-dimethyl-7-methylene	
23.480	3,10-Diazatricyclo[5,2,1.O(2,6)].4-diene, 3-ethenyl-10-ethyl	
23.625	1-(3,4-Dimethylphenyl)-2,5-dihydro-1H-pyrrole-2,5-dione	
23.706	p-Nitroaniline	
23.801	Pyrimidin-2-amine. N-(imino)(morpholino)methyl-4,6-dimethyl	

23.920	N-Cyclohexanecarbonylanthranilic acid	
24.000	4-Fluro-4’-hydroxybenzophenone	
24.044	3,5-Methano-2H-cyclopenta[b]furan-2,4(5H)-dione, 3,3a,6,6a-tetrahydro-, (3r,3a-trans,5-cis,6a-trans)-	
24.446	Nonane, 5-butyl-	
24.623	Isoquinolinium, 2-[(aminocarbonyl)amino]-hydroxide.inner salt	
24.753	N-methylanilino-1,3-butadien-1-yl)-4-(methoxycarbonyl)	
24.967	4a,5,6,7,8,9,9a-octahydro-3,5,6-trimethyl-9-methylene	
25.041	(3aS, 6aS)-3,3-dimethyl-3,4,5,6-tetrahydro-3a,6a-methanopentalen-1(2H)-one	
25.160	2-Butyl-1-iodo-bicyclo[2,2.1]heptanes	
25.229	4,5,6,6a-Tetrahydro-2(1H)-pentalenone	
25.362	1,2-Cyclopentanedicarboxylic acid, 4-(1,1-dimethylethyl)-, dimethyl ester, (1.alpha.,2.alpha.,4.alpha.)	
25.454	3,3-(2,6-Dioxocyclohexylidene)diproplonitrile	
25.593	Isophthalic acid, di(3,4-dimethylcyclohexyl) ester	
25.642	3-Methyllselenomethylfuran	
25.773	Acethydrazide, 2-(4-fluorophenoxy)-N2-(4-allyloxybenzylideno)-	
25.961	Ethyl cyanoglyoxylate-2-oxime	
26.001	Adipic acid, diphenyl ester	
**26.559**	**Heptadecane, 2-methyl** [11]	
**29.692**	**Octadecane, 1-chloro** [11]	
26.927	Benzenemethanol, 2,4-dichloro	
27.280	Oxalic acid, dodecyl isobutyl ester	
27.666	2-butenamide, N,N-diethyl-2-methyl-, (E)	

28.817	1H-Pyrrole, 1-(4-methylphenyl)-	
29.692	pronene, 1-chloro-	
30.019	1,8-Bis(allyloxymethyl)naphthalene	
32.112	2-Methylenecyclohexanol	
32.508	Terephthalic acid, di(2-ethylhexyl) ester	
35.243	2-Methyl-3-phenyl-pyrrolo(2,3-b)pyrazine	
**Dichloro methane Extracts**		
**19.897**	**Nonadecane** [11]	
19.967	Phthalic acid, di-(1-hexen-5-yl)ester	
20.327	s-Triazolo[4,3-a]pyridine, 3-ethyl-5-methyl	
**20.705**	**Aristolene** [11]	
20.822	1,4-Methanoazulene decahydro-4,8,8-trimethyl-9-methylene-, [1S-(1.alpha.,3a.beta.,4.alpha.,8a.beta.)	
22.663	1,1’-Bicyclohexyl	
22.741	Carbonic acid, methyl 4-methylphenyl ester	
22.939	p-Pentyloxynitrobenzene	
23.400	2-Acetyl-4-methylpyridine	
24.057	Acetic acid, 10-dimethoxymethyl-13-methyl-3-oxo,5,6,7,8,9,10,11,12,13,14,15,16,17-tetradecahydro-3H-cyclopenta[a]phenanthren-17-yl (ester)	
24.453	Propanoic acid, 3-phenoxy-, methyl ester	
24.617	Cyclopropane, 1-bromo-2,2,3,3-tetramethyl-1-prop-1-ynyl	
**25.347**	**Eicosane** [12]	
**25.350**	**-**	**Dodecane, 2,6,10-trimethyl**
**25.352**	**Hexadecane** [11]	
**26.596**		**12-Octadecenoic acid, methyl ester** [12]
27.634		2-Hexen-4-yn-1-ol, (E)-

27.738		3,7-Dimethyl-8-1,5-dioxa-spiro[5.5]undecane-3-carb2-Hexen-4-yn-1-ol, (E)-
**27.959**	**Octacosane** [14]	
27.963		1-Cyclohexanol, 4-tert.butyl-1-methyl
29.543		Tetracosahexaene, 2,6,10,15,19,23-hexamethyl
32.380		Hexanedioic acid, mono(2-ethylhexyl)ester
	**Ethyl acetate Extracts**	
10.232		2-Dodecene, (E)
10.527	-	Heptane, 3-methylene
15.409	2,5-Dimethylhexane-2,5-dihydroperoxide	
**16.076**		**2-Tetradecene, (E)-** [15]
**16.093**	**2-Tetradecene, (E)** [15]	
16.498		1,1-Dimethyl-1-silacyclo-3-pentene
17.626		Cyclohexadiene-1,4-dione,2,6-bis(1,1-dimethylethyl)-
**18.481**		**Phenol, 2,4-bis(1,1-dimethylethyl)-**
**18.482**	**Phenol, 2,4-bis(1,1-dimethylethyl)-**	
19.689		1, 13-Tetradecadien-3-one
**19.788**	**1-Hexadecene** [11]	
**20.083**		**1-Hexadecene** [11]
20.172		2-Propyn-1-amine, N,N-diethyl-
22.650		3-Cyclopentylpropionic acid, 3-methylbutyl ester
**22.724**		**E-15-Heptadecenal**
**22.726**	**E-15-Heptadecenal**	
22.919	2-Cyclopropylcarbonyloxytetradecane	
23.102		Benzenamine, 2,6-dimethyl-

24.317		Syn-cis-tricyclo[7.3.0.0(2,6)]dodecane, (+-)(7S, 8R)-
24.440		7,9-Di-tert-butyl-1-oxaspiro(4,5)deca-6,9-diene-2,8-dione
24.445	1-Phenanthrylene oxide	
24.554		Z-6,17-Octadecadien-1-ol acetate
24.655		1-Methyl-2-methylene-trans-decalin
**24.658**	**18-Norabietane**	
24.915		Exo-tricyclo[5.2.1.0(2,6)]decane
24.960	5-Decanone	
25.013	Oxepine, 2,7-dimethyl	
**25.128**		**18-Norabietane**
25.132	5,6,7,7a,9,10,11,12-Decahydrobenzo[b]fluoranthene	
25.215		3,7-Dimethyl-3-octyl methylphosphonofluoridate
25.278		**1-Octadecene** [12]
25.282	**E-15-Heptadecenal**	
25.420	11,13-Dimethyl-12-tetradecen-1-ol acetate	
25.464		5-methyl-2-thiophenecarboxaldehyde
25.824		E-8-Methyl-9-tetradecen-1-ol acetate
26.198		Trans-4-Choloro-4’-methoxychalcone
26.570		Heptafluorobutyric acid, n-octadecyl ester
26.648	Cyclohexane, methylene	
26.665		Propane, 1,1,1,2,2,3-hexachloro-3,3-difluoro-

27.051		2-Pyridinemethanol, 5-ethoxy-
27.871		1-Docosene
**29.748**		**Eicosane** [12]
27.877	Trichloroacetic acid, pentadecyl ester	
29.819		1-Ethyl-4-methylcyclohexane
29.848	3-Methyl-2,3-dihydro-1,5-benzothiazepin-4(5H)-one 1-oxidex	
31.947		Heptadecylpentafluoropropionate
31.950	Trichloroacetic acid, tridecyl ester	
32.084		**Heptadecane** [11]
35.199		Tetratetracontane
